# Principal component analysis–artificial neural network-based model for predicting the static strength of seasonally frozen soils

**DOI:** 10.1038/s41598-023-43462-7

**Published:** 2023-09-26

**Authors:** Yiqiang Sun, Shijie Zhou, Shangjiu Meng, Miao Wang, Hailong Mu

**Affiliations:** 1 College of Civil Engineering and Architecture, Harbin University of Science and Technology, Harbin, 150080 China; 2 Key Laboratory of Earthquake Engineering and Engineering Vibration, Institute of Engineering Mechanics, China Earthquake Administration, Harbin, 150080 China; 3 School of Architecture and Civil Engineering, Heilongjiang University of Science and Technology, Harbin, 150022 China; 4 College of Architecture and Civil Engineering, Heilongjiang Province Hydraulic Research Institute, Harbin, 100050 China

**Keywords:** Civil engineering, Natural hazards

## Abstract

Seasonally frozen soils are exposed to freeze‒thaw cycles every year, leading to mechanical property deterioration. To reasonably describe the deterioration of soil under different conditions, machine learning (ML) technology is used to establish a prediction model for soil static strength. Six key influencing factors (moisture content, compaction degree, confining pressure, freezing temperature, number of freeze‒thaw cycles and thawing duration) are included in the modelling database. The accuracy of three typical ML algorithms (support vector machine (SVM), random forest (RF) and artificial neural network (ANN)) is compared. The results show that the ANN outperforms the SVM and RF. Principal component analysis (PCA) is combined with the ANN, and the PCA–ANN algorithm is proposed, which further improves the prediction accuracy. The deterioration of soil static strength is systematically researched using the PCA–ANN algorithm. The results show that the soil static strength decreased considerably after the first several freeze‒thaw cycles before the strength plateau occurred, and the strength reduction increased significantly with increasing moisture content and compaction degree. The PCA–ANN model can generate a reasonable prediction for the static strength or other soil properties of seasonally frozen soil, which will provide a scientific reference for practical engineering.

## Introduction

Seasonally frozen soil refers to soil that freezes in winter and completely melts in summer, usually within a few metres from the ground^1^. Regions where there is seasonally frozen soil cover are referred to as seasonally frozen areas. Soils in these regions will be exposed to freezing and thawing many times every year, especially those that are just beneath the surface. The properties of the soil will change significantly after freeze‒thaw cycles, which is one of the leading causes of engineering problems in seasonally frozen regions, and this has been widely recognized by researchers^2,3^. For this reason, it is significant to analyse the mechanical properties of seasonally frozen soil^2,4–6^.

At present, laboratory tests are the most frequently used method to research the freeze–thaw characteristics of seasonally frozen soils. During the last few decades, scholars have conducted extensive investigations on the mechanical properties of frozen–thawed soils and summarized the variation rules of different soil mechanical properties under varying conditions. The results show that the compressive strength^7^, cohesion^1^, friction angle^8^ and other soil properties are greatly changed after freezing and thawing and that the variations in soil characteristics are affected by many factors, such as the freezing temperature^9^, strain rate^10^ and number of freeze‒thaw cycles^11–13^. However, these experiments are costly and time-consuming, especially when the number of freeze‒thaw cycles is large. To reduce the number of exhaustive tests, some mathematical formulas for predicting the soil properties were proposed based on the limited testing results. It is necessary to consider multiple factors simultaneously to obtain an accurate prediction model. At present, most of these prediction models are established by fitting the experimental data directly^14–16^. However, it is extremely difficult or even impossible to comprehensively consider all of these influencing factors in this way. Furthermore, problems such as tedious derivation processes and inconvenient implementations also limit the application and development of this method.

In recent years, machine learning (ML) algorithm-based methods have gradually emerged and developed rapidly in various fields^17–22^. These ML-based methods also have great advantages in solving different modelling and prediction problems encountered in geotechnical engineering^23–29^. For instance, Esmaeili-Falak et al^30^. compared the performance of three ML algorithms in predicting the mechanical properties of frozen soil. The results indicated that all three ML models established were reliable in terms of predicting the mechanical properties of frozen soil. Benemaran et al^31^. used a variety of ML algorithms to model and predict the Young 's modulus of frozen sand. The prediction results show that hybrid Additive Regression-Gaussian Process Regression and Bagging-Gaussian Process Regression have the best accuracy. Das et al^32^. utilized the ANN and SVM for predicting the swelling pressure of soil and found that the SVM achieved superior performance. Moreover, Das et al^33^. arrived at a similar conclusion when predicting the mechanical parameters of cemented soil. The superiority of using ML algorithms to predict the mechanical properties of rock and soil is as follows. First, unlike other traditional methods of tedious theoretical derivation, ML algorithms can directly find the internal relationship between testing results without any presupposed hypothesis, so the modelling is easier to conduct. Then, the ML-based prediction models can consider as many influencing factors as possible simultaneously. Thus, the predicted values are in close agreement with the experimental values. Finally, the accuracy of the model based on ML algorithms can be continuously improved with increasing experimental data. If the training set is sufficiently large, the predicted values will be almost identical to those tested. Due to the above strengths, an increasing number of scholars have begun to apply ML algorithms to solve the nonlinear problems faced in geotechnical engineering. There are many kinds of algorithms to choose from when building an ML prediction model, such as artificial neural networks (ANNs)^25,34,35^, decision trees (DTs)^26^, random forests (RFs)^36,37^, support vector machines (SVMs)^38^, and evolutionary polynomial regression (EPR)^39^. The prediction precision may be significantly different when various algorithms are employed, even with the same dataset^40^. Therefore, it is necessary to compare the performance of different algorithms to obtain satisfactory prediction results.

Although there have been many attempts to use ML technologies in geotechnical engineering, the application of ML in predicting the mechanical properties of soil before and after freezing–thawing cycles has not been reported in the published literature. To accurately predict the static strength of freeze‒thaw soil while accounting for the influence of multiple factors and avoiding cumbersome formula derivations, a machine learning-based predictive model for freeze‒thaw soil strength is established in this paper. Various ML algorithms were used to predict the static strength (S) of the freeze–thaw soil. The influence of the number of freeze‒thaw cycles (N_FT_), thawing time (T_T_), negative temperature (T_N_), water content (*w*), compaction degree (*k*) and confining pressure (C_P_) on the static strength was considered. Three popular ML algorithms, namely, the RF, SVM and ANN, were selected to build a prediction model for the static strength of freeze–thaw soil. By comparing the accuracy of three models based on different ML algorithms, the algorithm with the highest precision was determined. In addition, the principal component analysis (PCA) algorithm was used to optimize the data structure, which further enhanced the prediction accuracy. Finally, the ML model based on PCA–ANN was used to predict the deterioration of soil static strength after freezing and thawing, and the influence of moisture content, compaction degree and confining pressure were analysed and discussed.

## Theory and methodology

### Support vector machine (SVM)

The SVM is a kind of supervised learning algorithm that is based on the structural risk minimization principle rather than the traditional empirical risk minimization principle^41^. The principle of the SVM is that the input data are first mapped to the high-dimensional space, and then linear regression is performed on the data in the high-dimensional space to solve the original nonlinear problem. For the original dataset, the nonlinear regression can be transformed into a linear fitting problem in the high dimensional space by the mapping function *ϕ*(*x*) in the SVM. The linear function is expressed as follows:

1 $$f(x) = \omega^{{\text{T}}} \phi (x) + b$$

where *ω* is the weight vector and *b* is the bias coefficient.

Unlike the traditional regression model, a certain gap between the predicted and actual values is allowed in the SVM. Only when the error exceeds the permissible value will it be regarded as a loss. The optimization objective function is as follows:

2 $$\mathop {\min }\limits_{\omega ,b} \frac{1}{2}\left\| \omega \right\|^{2} + C\sum\limits_{i = 1}^{m} {l_{\varepsilon } \left( {f(x_{i} ) - y_{i} } \right)}$$

where *C* is the regularization constant, *l*_*ε*_ is the epsilon insensitive loss function and $\parallel \omega \parallel^{2}$ is the Euclidian norm of the weight vector.

In the process of solving the above equation, the relaxation factor and Lagrange multiplier are introduced. The final regression model is as follows:

3 $$f(x) = \sum\limits_{i = 1}^{u} {\left( {\alpha_{i} - \alpha_{t}^{ * } } \right)K(x,x_{i} ) + b}$$

where *α*_*i*_ and* α*_i_^*^ are the Lagrange multipliers and $K\left( {x,x_{i} } \right) = \phi (x) \cdot \phi \left( {x_{i} } \right)$ is the kernel function. The kernel function is used to avoid the calculation on the high-dimensional characteristic space $\phi (x)$ and reduce the computational complexity.

### Random forest (RF)

The most commonly used integration algorithms include bagging and boosting, among which the RF is the most representative bagging algorithm^42^. Bagging is random sampling in the training set with a drop back, during which each sample is selected with equal probability. After extracting the same number of samples as that in the original dataset, a new dataset is formed. The RF is composed of multiple decision trees, which are built from a new dataset obtained by the bagging method. The final result of an RF is the average of all decision trees, which makes the predicted results more accurate. Supplementary Fig. 1 shows a schematic diagram of the formation of a random forest.

### Artificial neural network (ANN)

An ANN is a kind of feedforward neural network, and it is currently one of the most popular neural networks. In an ANN, neurons connect to each other end to end in a straightforward and intuitive way to form a network without closed rings. The output of one neuron is the input of another neuron. The topology of this model is shown in Supplementary Fig. 2.

The neural network is organized by layers, which can be divided into the input layer, hidden layer and output layer, according to their functions. The number of neurons in each layer should be determined by the input, output and accuracy requirements. The output of a neuron is composed of two parts: one is the linear combination of the values of the previous layer, and the other is the nonlinear transformation of the activation function. Supplementary Fig. 2b illustrates the processing of neurons. The weight (*w*) and bias (*b*) parameters in the linear model are continuously updated according to the output error when the network is established. One update round is referred to as an iteration, and the iterations will not stop until the ending rule is satisfied. In the process of nonlinear transformation, different activation functions can be selected to adapt to the different data structures.

### Principal component analysis (PCA)

PCA is a kind of descending dimension algorithm that can map high-dimensional data to low-dimensional space and make the data in low-dimensional space retain as much information as possible. Although the dimensionally reduced data will inevitably lose some information, it can enable us to better grasp the main features. In a low-dimensional space, there is more explanatory significance between the noncorrelated information. The main procedures of PCA are as follows:

An m × n order matrix of the experimental data is constructed and normalized (see Eq. 4).

4 $${\varvec{X}}_{m \times n} = \left[ {\begin{array}{*{20}c} {x_{11} } & {x_{12} } & \cdots & {x_{1n} } \\ {x_{21} } & {x_{22} } & \cdots & {x_{2n} } \\ \vdots & \vdots & \ddots & \vdots \\ {x_{m1} } & {x_{m2} } & \cdots & {x_{mn} } \\ \end{array} } \right] = \left[ {{\varvec{x}}_{1} ,{\varvec{x}}_{2} , \cdots ,{\varvec{x}}_{n} } \right]$$

where *m* is the number of samples and *n* is the number of features. Then, the covariance matrix of ***X*** is calculated and normalized as Eq. (5). Next, the eigenvalues and eigenvectors of the normalized covariance matrix R are calculated. The eigenvalues λ_i_ are arrayed in descending order: λ_1_ ≥ λ_2_ ≥ λ_3_ ≥ ··· ≥ λ_n_. The feature vectors *e*_*i*_ are expressed as Eq. (6).

5 $${\varvec{R}}_{n \times n} = \left[ {\begin{array}{*{20}c} {r_{11} } & {r_{12} } & \cdots & {r_{1n} } \\ {r_{21} } & {r_{22} } & \cdots & {r_{2n} } \\ \vdots & \vdots & \ddots & \vdots \\ {r_{n1} } & {r_{n2} } & \cdots & {r_{nn} } \\ \end{array} } \right]$$

6 $${\varvec{e}}_{1} = \left[ {\begin{array}{*{20}c} {e_{11} } \\ {e_{21} } \\ \vdots \\ {e_{n1} } \\ \end{array} } \right],\;{\varvec{e}}_{2} = \left[ {\begin{array}{*{20}c} {e_{12} } \\ {e_{22} } \\ \vdots \\ {e_{n2} } \\ \end{array} } \right], \ldots ,{\varvec{e}}_{n} = \left[ {\begin{array}{*{20}c} {e_{1n} } \\ {e_{2n} } \\ \vdots \\ {e_{nn} } \\ \end{array} } \right]$$

The proportion of information extracted from a principal component to the total information is defined as the contribution rate of the explicable variance. For example, the contribution rate of the *i*th principal component is:

7 $$C = \frac{{\lambda_{i} }}{{\sum\limits_{k = 1}^{n} {\lambda_{k} } }}$$

Finally, the principal component *y*_*i*_ is calculated:

8 $$y_{i} = e_{1i} x_{1} + e_{2i} x_{2} + \cdots + e_{ni} x_{n}$$

### Framework of the model in this work

The original data have different units whose influences cannot be identified in the analysis, and the effect of factors with large absolute values will be highlighted. For this reason, the initial data were standardized to eliminate the impact of dimension.

Supplementary Fig. 3 shows the establishment of the ML model for predicting the static strength of seasonally frozen soil. The data were normalized before being input into the model, so the impact of dimension was eliminated. After that, the data were divided into three parts, among which 70% (86) of the data were used for training the model, 20% (22) were used for verification, and the remaining 10% (12) were used to test the generalizability of the established model. Such a partition makes the ratio of training data to the validation data nearly 8:2 (86:22), which can ensure that the ML-based model is well trained and tested, as has been proven theoretically^43^. The division of the data set is achieved by using the split function in Python. Then, models with different ML algorithms were built based on the training data, and the hyperparameters of these models were optimized according to their performance on the validation set. Methods used for the optimization of hyperparameters include grid search, random grid search and learning curve. After determining each of the optimal models established using the RF, SVM and ANN, these three models were compared in terms of their performance on the validation set, and the best model was selected. The chosen model was combined with the PCA algorithm to further optimize its performance. Finally, the generalizability of this model was evaluated by the predicted results of the testing set. Data in the testing set are never seen by the model, so it is reasonable to evaluate the model’s generalizability on the testing set.

## Data collection and ML models

### Data collection

A series of triaxial tests were conducted on the frozen-thawed soil specimens in the author’s previous work^44^, and a dataset containing 120 groups of experimental data on the static strength of soil was created. The effects of six factors, namely, moisture content, compaction degree, confining pressure, freezing temperature, freezing times and thawing duration, on the static strength were considered. The data set used in this paper is given in Supplementary Table 2, and statistical specifications for the dataset have been calculated as shown in Supplementary Table 3. The input and output distribution histograms and violin plots in the dataset are shown in Supplementary Fig. 4–7. In order to better observe the relationship between the input features, Supplementary Fig. 8 gives the Pearson Correlation Curve heat-map of the input data. Except for the static strength, which was being tested and used as the target variable, all of the other items were treated as input variables when building the model.

### Optimization of hyperparameter

The three models in this section were all implemented using the sklearn ML algorithm library in Python. The hyperparameters of the three different algorithms were optimized and selected using the methods of grid search, random grid search and learning curve.

The grid search method refers to the traditional enumerated grid search. However, when the parameter space or data volume is too large, the time for grid searching will increase significantly. Therefore, there are two main methods to optimize the grid search correspondingly. One is to restructure the search space, and the other is to adjust the data of each training. The random grid search is the first kind of method. The specific way of adjusting the parameter space is to abandon the global hyperparameter space in the original search and select some parameter combinations instead. A hyperparameter subspace is constructed using these selected parameter combinations, and the search is only implemented in this subspace. In this way, the search can be expanded under the same number of searches, and the operation speed can be accelerated within the same search range. Furthermore, the minimum losses of the two methods are nearly the same. The schematic diagrams of the two search methods are shown in Supplementary Fig. 9.

The principle of the learning curve is to draw the line of model scores with different values of hyperparameters so that the optimal hyperparameter can be found in a visual manner. In addition, the variance of the model performance with the change of a specific parameter can be shown in the learning curve, which prevents the continuously increasing parameter but with slightly improved model performance. The hyperparameters selected for the three algorithms are listed in Table 1, and how to determine them is described in detail below.

1. For the SVM algorithm, the kernel function, C, and gamma were selected as the hyperparameters. The linear kernel (linear), polynomial kernel (ploy), hyperbolic tangent kernel (sigmoid) and Gaussian radial basis kernel function (RBF) are four commonly used kernel functions. Since the Gaussian radial basis kernel can adapt to most data, the RBF was chosen as the kernel function. C is the penalty parameter, which represents the tolerance of the model to the error. A larger C means that the model is less likely to have errors and is prone to overfitting. On the other hand, a smaller C reveals that the model is more tolerant to errors and that it is prone to underfitting. Gamma, a parameter of the kernel function, has an important impact on the robustness and accuracy of the model. First, C and gamma were searched in a large scope through the random grid search, and then a precise search was performed by the grid search in a shrinking range. The ranges of the search are shown in Supplementary Table 4. It is found that the value of gamma is basically stable, but C is constantly increasing. Therefore, the learning curve is used to observe the trend of R^2^ with the increase in C under the optimal gamma, and the result is depicted in Supplementary Fig. 10a. Supplementary Fig. 10a shows that when C exceeds 150, the performance of the model gradually tends to stabilize. To ensure the generalizability of the model, C and gamma were set as 150 and 0.77, respectively.
2. For the RF algorithm, the parameter of n_estimators was adjusted and determined. This parameter represents the number of populations in the forest. The properties of random forest cannot be displayed well when the n_estimators is too small. However, the prediction accuracy of the model will not be improved continuously with increasing n_estimators, and the run time will increase dramatically if the n_estimators are beyond a certain value. Supplementary Fig. 10b shows the change in R^2^ with the increase in trees, and this parameter was finally set as 338. The results on the testing set and training set show that there is no overfitting phenomenon, so the tree does not need to be cut. Other parameters are set as the default values.
3. For the ANN algorithm, three key parameters, the solver, activation function and hidden_layer_sizes, were chosen to investigate their influence. The default settings of sklearn were used for the other parameters. The solver represents the calculation method for parameters in the ANN, including stochastic gradient descent (SGD), adaptive moment estimation (Adam) and limited-memory Broyden–Fletcher–Goldfarb–Shanno (LBFGS)^45^. There are four types of activation functions in sklearn: ‘identity’, ‘logistic’, ‘relu’ and ‘tanh’.

**Table 1:** Optimal hyperparameters for different algorithms.

Algorithms	Hyperparameters	Value
SVM	Kernel	rbf
	C	150
	Gamma	0.77
RF	N_estimators	338
ANN	Solver	lbfgs
	Activation function	relu
	Number of the hidden layer	7

In most models, one hidden layer can meet the requirements for accuracy, so the number of hidden layers is set to one here. However, there has been no consensus on the optimal number of nodes in the hidden layer, so its approximate range is usually determined by the empirical formula shown in Eq. (9).

9 $$m = \sqrt {n + l} + \alpha$$

where *m* and *n* are the number of nodes in the hidden and input layers, respectively, *l* is the number of nodes in the output layer, and *α* is a constant between 1 and 10.

The solver and activation function were determined by the traditional enumerated grid search. The optimal one was obtained by enumerating all combinations, and the final two hyperparameters were ‘LBFGS’ and ‘RuLU’ for the solver and activation function, respectively. First, the range of 3–14 was determined by the empirical formula. However, the scope was expanded to 3–20 to ensure that the optimal number of nodes was chosen. The learning curve of R^2^ to the number of nodes is illustrated in Supplementary Fig. 10c, and the number was finally set as 7.

### Evaluation of the model performance

The optimal hyperparameters corresponding to each model based on different algorithms were determined after the above optimization, and then three models were established with these hyperparameters. The performance of each model was assessed by the following indicators: coefficient of determination (R^2^), mean absolute error (MAE), mean square error (MSE) and root mean square error (RMSE). The prediction accuracy of these models can be revealed objectively by the parameters mentioned above from different aspects.

R^2^ is calculated as Eq. (10), reflecting the fitting degree of the regression line to the observed value. The closer to 1 this value is, the better the fit.

10 $$R^{2} = \frac{SSR}{{SST}} = \frac{{\sum\limits_{i = 1}^{n} {\left( {\hat{y}_{i} - \overline{y}} \right)^{2} } }}{{\sum\limits_{i = 1}^{n} {\left( {y_{i} - \overline{y}} \right)^{2} } }}$$

where $\hat{y}_{i}$ is the predicted value, $y_{i}$ is the experimental data and $\overline{y}$ is the average value of $y_{i}$.

The MAE is the mean value of the absolute error, reflecting the amplitude of the average error. A smaller MAE means a better prediction.

11 $${\text{MAE}} = \frac{1}{n}\sum\limits_{i = 1}^{n} {\left( {\left| {y_{i} - \hat{y}_{i} } \right|} \right)}$$

*n* is the number of samples, and $y_{i}$ and $\hat{y}_{i}$ are the same as those above.

The MSE is the expected value of the square of the error, and it reflects the sum of squares for the deviation between the observed and actual values.

12 $${\text{MSE}} = \frac{1}{n}\sum\limits_{i = 1}^{n} {\left( {y_{i} - \hat{y}_{i} } \right)^{2} }$$

The RMSE is the square root of the MSE and is calculated via Eq. (13).

13 $${\text{RMSE}} = \sqrt {\frac{1}{n}\sum\limits_{i = 1}^{n} {\left( {y_{i} - \hat{y}_{i} } \right)^{2} } }$$

### Model comparison and algorithm selection

The performance of the three models on the training and testing set are compared, and the results of the *R*^2^, MAE, MSE and RMSE are shown in Table 2, and provided model rankings for each parameter in Supplementary Table 5. To observe the performance of different models more intuitively, Supplementary Fig. 11 gives the Taylor diagrams of three models.

**Table 2:** Performance evaluation of different algorithms.

	SVM		RF		ANN	
	Training set	Validation set	Training set	Validation set	Training set	Validation set
MSE	71.7	267.3	36.9	234.9	66.1	141.3
MAE	5.0	9.4	3.9	8.2	5.7	8.1
RMSE	8.5	16.3	6.1	15.3	8.1	11.9
*R*^2^	0.97	0.92	0.98	0.93	0.97	0.96

It can be seen from Table 2 that the performance of each model on the training set is better than that on the validation set, but the gap between them is not obvious, which proves that there is no overfitting phenomenon. Then, the situation of different models on the testing set was compared, and the fitting to the predicted data and the value of error revealed that the ANN algorithm outperformed the other two. Although the performance of the RF was even better than that of the ANN on the training set, it was far from that of the ANN on the validation set, indicating that the generalizability of this model is poor. The result of the SVM is similar to that of the RF.

Supplementary Fig. 12 shows the comparison between the tested and predicted results on the validation set of the three models. It can be seen that most of the errors of these three algorithms can be controlled within a narrow range. However, individual results show a relatively large error, which is more obvious in the RF and SVM algorithms. This can be explained by the small amount of data and the inaccurate measurement of some experimental data and can be improved by increasing the training data and enhancing the experimental precision. For example, for the 6th test, all three algorithms have similar errors, which is sufficient to indicate that there may be some imperfection for this single experiment, so it is important to improve the testing accuracy.

In conclusion, among the three kinds of algorithm models, the ANN is the best option for the problem studied in this paper. In addition, if the accuracy and quantity of experimental data are increased, the prediction ability of ML algorithms will be further improved.

## Optimization of the ANN model

According to the discussion in previous Section, the ANN is the most suitable algorithm to predict soil strength behaviours after freezing and thawing. In this section, the ANN is combined with the PCA algorithm to optimize the network and the data structure, aiming to further improve the prediction precision. As a tool for data structure processing, PCA can reduce the dimension of the data and compress the data. Meanwhile, the correlation between data can be reduced, making it easier for the model to capture important information in the data.

### PCA–ANN model

The number of features retained in the data after dimensionality reduction was determined by the cumulative explained variance before conducting the PCA processing. It is considered reasonable when the accumulative variance reaches 85%. Supplementary Fig. 13 shows the corresponding explainable variance and its cumulative value of each principal component. The data after dimensionality reduction accounted for 89% of the original data when four features remained and 95% when five features were retained. To retain more data information while achieving dimensionality reduction, five principal components were retained here.

After PCA dimensionality reduction of the data, the hyperparameters of the model were adjusted again. The selection of hyperparameters was basically the same as the adjustment to the neural network in the previous section, so it will not be repeated here. Supplementary Fig. 14 shows the R^2^ of models with different numbers of nodes in the hidden layers. Finally, ‘activation: tanh’ and ‘solver: LBFGS’ were selected as hyperparameters, and the number of nodes in the hidden layer was 12.

### Precision evaluation before and after PCA processing

The prediction results before and after PCA processing were compared, and the four evaluation parameters for the ANN and PCA–ANN are listed in Table 3. For the testing set, the performance of the model after PCA processing is better than that of the ANN. Although the accuracy on the testing set is only slightly improved, there is no doubt that the generalizability of the PCA–ANN algorithm is superior to that of the ANN.

**Table 3:** Comparison of the model performance before and after PCA.

	ANN		PCA–ANN	
	Training set	validation set	Training set	validation set
MSE	66.1	141.3	73.8	115.0
MAE	5.7	8.1	6.1	7.7
RMSE	8.1	11.9	8.6	10.7
R^2^	0.97	0.96	0.97	0.97

The prediction errors of the models before and after PCA processing are compared in Supplementary Fig. 15. Although the model combined with the PCA algorithm does not reduce the error of each prediction result significantly, the overall prediction precision is improved, which verifies the principle of the PCA algorithm again. Although the network has not been significantly optimized, the prediction accuracy has been improved, indicating that the data structure has been optimized and adjusted. If there are more input nodes, the optimization of the network structure will become obvious. In summary, the PCA–ANN algorithm has superior performance to the ANN.

### Assessment of the generalizability

**Figure 1:**
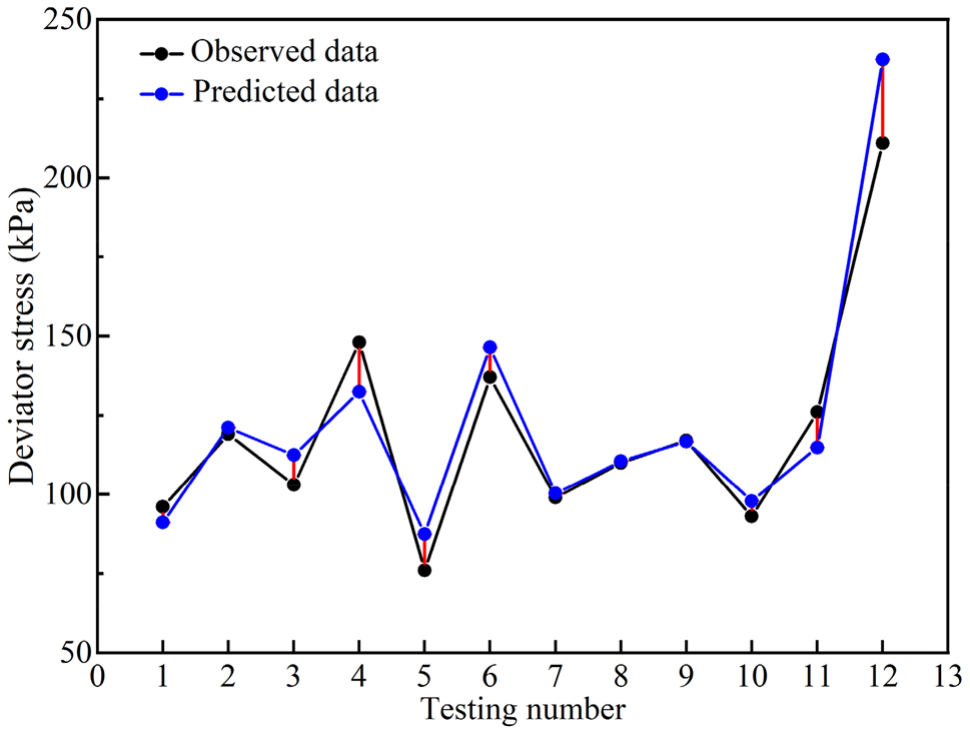
Prediction results on the testing set.

The generalizability of the model was evaluated through the 10% raw data, which was not used for training or optimizing the model previously. The results show that the R^2^ of the testing set is 0.90, the RMSE is 10.8, and the MAE and MSE are 8.1 and 118.7, respectively, indicating that the prediction results of the model on the testing set are also satisfying. Figure 1 shows the gap between the tested and predicted data, which demonstrates that the model is reasonable in depicting the characteristics of the unseen data. The error of most points was within 10%. For the large errors of some single points, the reason is similar to those analysed above, so it will not be repeated here.

The above results demonstrate that the model has satisfactory precision and strong generalizability, which can meet the requirements for predicting the properties of soil after freeze–thaw cycling. In practice, more accurate results will be obtained with the enlarged experimental dataset and improved precision in the tested results.

### Sensitivity analysis

**Figure 2:**
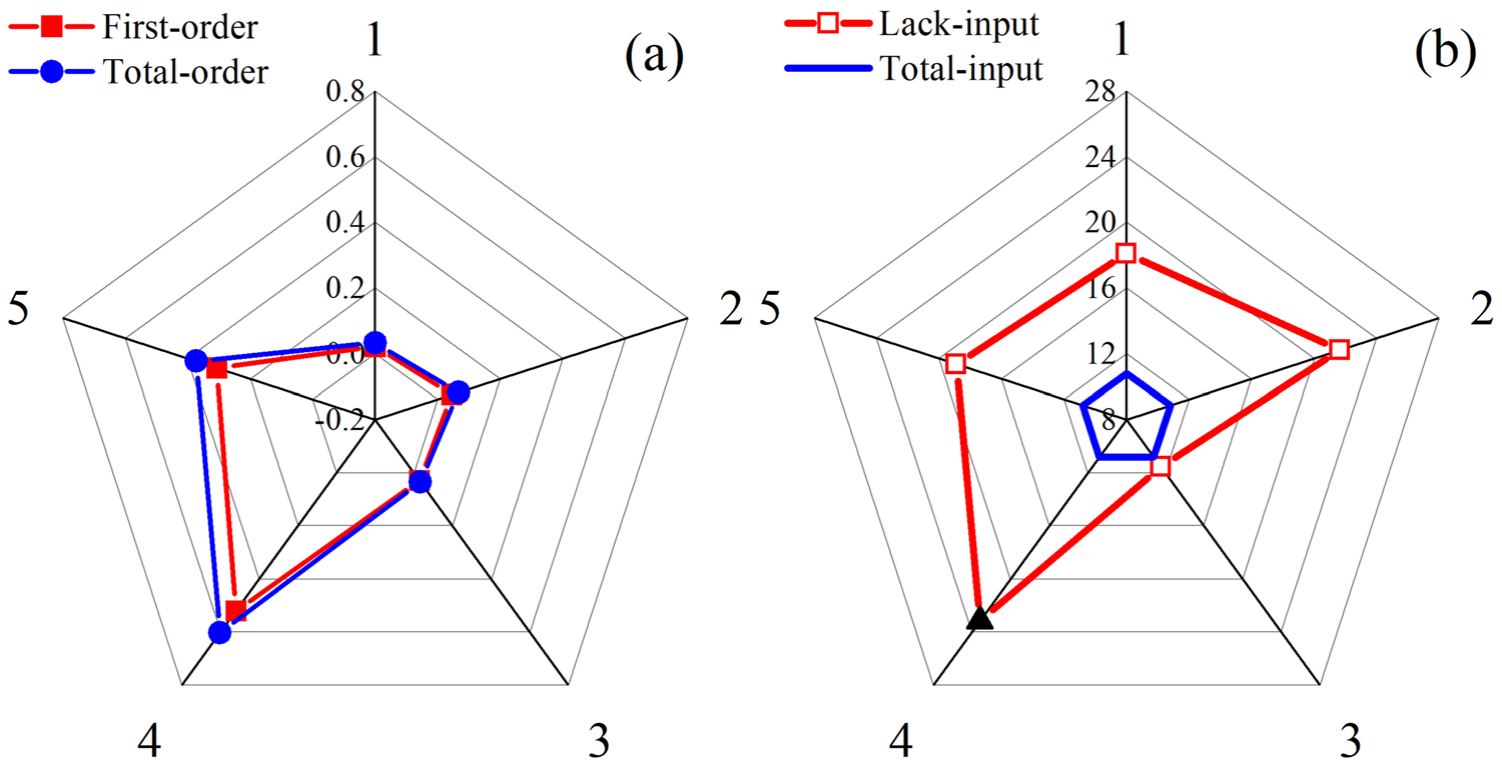
Model sensitivity analysis index and its different input error plots (**a**) Si, (**b**) RMSE.

Sensitivity analysis was conducted on the five principal components obtained through PCA. It computed both the first-order sensitivity indices and total-order sensitivity indices (Si) for each principal component, as illustrated in the Fig. 2a. The results indicate that principal component 4 exhibits the highest first-order sensitivity index and total-order sensitivity index, reaching 0.52 and 0.6, respectively. This implies that principal component 4 has a significant impact on the model output and exerts substantial influence even when interacting with other variables. Furthermore, to validate these findings, different training sets were created by systematically removing one input parameter at a time. The root mean square errors (RMSE) for various testing set were computed, as depicted in the Fig. 2b. Notably, the RMSE increases most significantly when principal component 4 is removed, which further proves the significant sensitivity of principal component 4 in the model.

## Predictions of the deterioration of seasonally frozen soil

According to the discussion in the previous sections, the ML model established by the PCA–ANN algorithm can provide a comparatively accurate prediction for the static strength of the frozen-thawed soil. The freeze‒thaw cycles change the structure of the soil, so the physical and mechanical properties of the soil continuously deteriorate with each cycle. Therefore, it is necessary to study the deterioration law of frozen-thawed soil. In this section, a prediction model for the deterioration law under different conditions was established by the same method as that in the previous section. Forecasting in this section is the interpolated prediction for completely unknown levels, so the model needs to learn as much information as possible. For this reason, the model learned all of the data after the hyperparameters were determined. Finally, the prediction results were compared with the findings in Sun et al.^44^.

### Influence of moisture content

**Figure 3:**
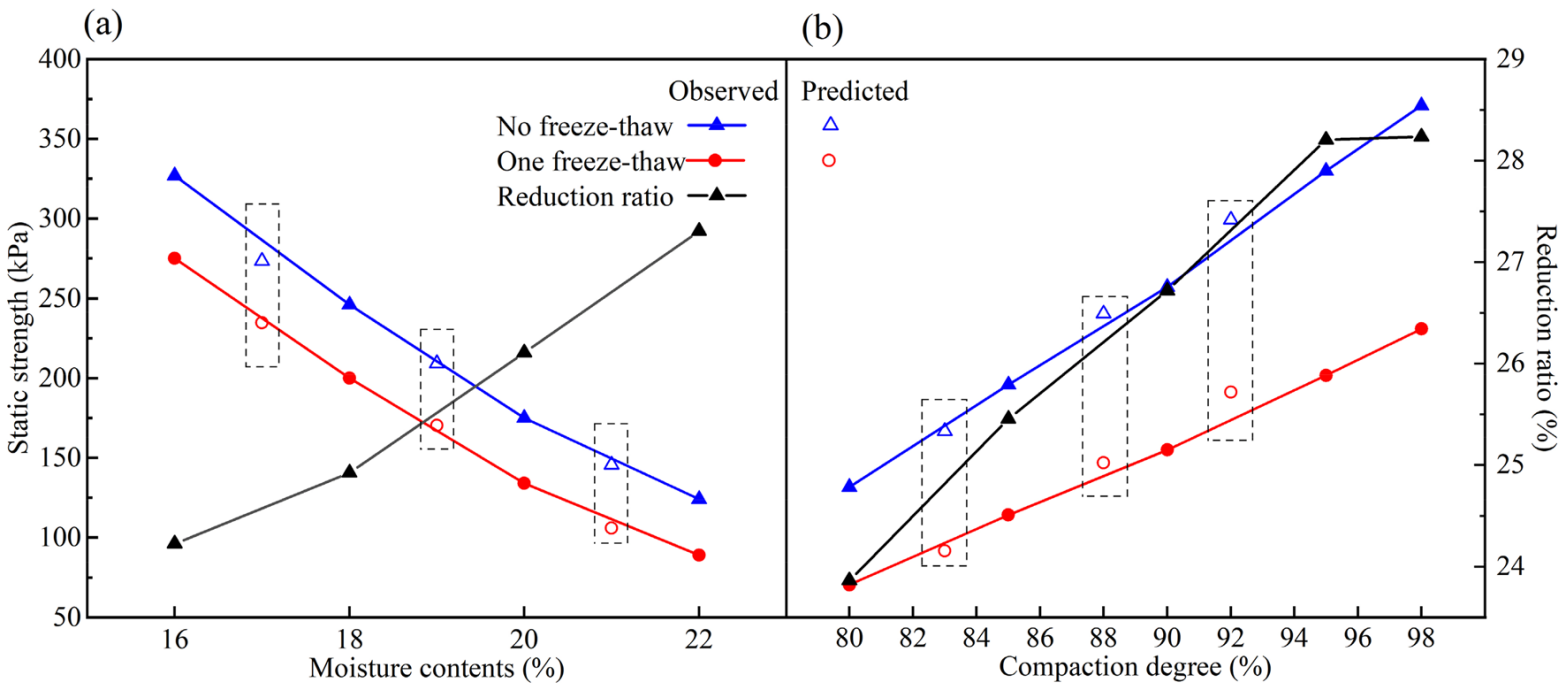
Static strength under different moisture contents and compaction degree (**a**) moisture contents, (**b**) compaction degree.

The deterioration of the static strength of the frozen-thawed soil with different moisture contents is shown in Fig. 3a. It can be easily found that the static strength of soil falls markedly after one freeze‒thaw cycle, which is true for all four kinds of experimental moisture contents. During the freezing process, the unfrozen water in the soil migrates to the freezing front continuously, causing the redistribution of water in the soil. The water inside the soil pores freezes into ice when the temperature decreases to the freezing point, and its volume expands, which leads to the failure of the soil structure, following the reduction in soil static strength. The volume expansion of soil due to frost heave increased with the moisture content, resulting in larger soil pores and decreased strength, so the strength reduction ratio increased with increasing moisture content.

The experimental conditions in Fig. 3a are fixed except for the moisture content. Unconsolidated–undrained triaxial tests were conducted on specimens with moisture contents of 16%, 18%, 20% and 22%, and the static strengths of specimens with moisture contents of 17%, 19% and 21% were predicted by the PCA–ANN algorithm. The interpolation prediction results are also depicted in Fig. 3a. It can be observed that the predicted results basically agree with the measured curves, which means that the degradation of soil static strength after freeze‒thaw cycling can be reasonably predicted even under untested moisture contents.

### Deterioration law under different compaction degrees

Figure 3b shows the deterioration of static strength for the frozen-thawed soil with compactness values of 80%, 85%, 90%, 95% and 98%. This illustrates that the static strength of the soil decreases after freeze‒thaw cycles, which is true for all of the tested specimens with different compaction degrees.

Unlike the effect of moisture content, the static strength of soil increases with increasing compactness, whether it is subjected to freeze‒thaw action. This suggests that the increase in compaction enhances the soil strength. However, the action of freezing and thawing has an opposite effect on the soil with a denser structure. That is, the attenuation ratio of the static strength caused by the freeze‒thaw cycle will increase with the compaction degree, which means that the strength deterioration after the freeze‒thaw cycle is more distinct for soil with a denser structure. This can be attributed to frost heave by the volume expansion of pore water during freezing. The denser the initial state of the soil is, the more obvious the volume change after freezing, following more serious structural damage and greater strength reduction.

Based on the PCA–ANN algorithm, the static strengths for specimens with compaction degrees of 83%, 88% and 92% were predicted, and the results are shown in Fig. 3b for comparison. It can be observed that the predicted results of the PCA–ANN algorithm conform to the experimental curve, namely, a denser specimen has a higher strength. The influence of compaction degree on the strength reduction of frozen-thawed soil is also observed from the prediction results. As the compaction degree increases, the gap between the static strength of the unfrozen and frozen-thawed soils gradually broadens. This illustrated that reasonable prediction results can be obtained for frozen-thawed soil with different compaction degrees.

### Variations in the static strength with the confining pressure and number of freeze‒thaw cycles

**Figure 4:**
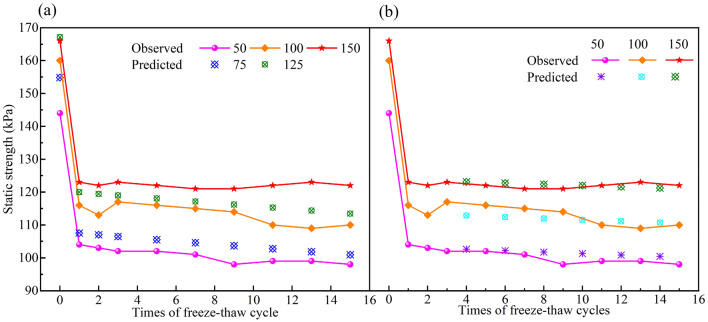
Static strength under different confining pressures and freeze–thaw cycles (**a**) confining pressures, (**b**) freeze–thaw cycles.

Unconsolidated–undrained triaxial tests with confining pressures of 50 kPa, 100 kPa and 150 kPa were conducted on the specimens that experienced different numbers of freeze‒thaw cycles. The deterioration of static strength under different cell pressures and freeze‒thaw cycles is shown in Fig. 4a.

Figure 4a shows that the static strength of soil decreases after freeze‒thaw cycles under different confining pressures, but the strength reduction tends to be restrained with increasing confining pressure, indicating that the strength deterioration declines under higher confining pressure. In a sense, the cell pressure can recover the freeze‒thaw microcracks and fissures by rearranging and consolidating the soil particles, so the strength of specimens will be enhanced under higher confining pressure^44^. The interpolated predictions are performed for confining pressures of 75 kPa and 125 kPa, and the results are depicted in Fig. 4a. The predicted static strength for specimens with given confining pressures of 75 kPa and 125 kPa is located in the zone formed by the two adjacent experimental lines, which reveals that the PCA–ANN algorithm is effective in simulating the strength properties of frozen-thawed soil under different cell pressures.

What is also evident from Fig. 4a is that there is a considerable drop in the soil static strength after the freeze‒thaw cycles, and a significant reduction in strength occurs in the first few freeze‒thaw cycles. Then, the strength reduction slowed until seven to nine cycles were reached, after which the strength gradually tended to be constant. To verify the feasibility of PCA–ANN on the deterioration prediction of seasonally frozen soil under cycles of freezing and thawing, the static strength of specimens that experience 4, 6, 8, 10 and 12 freeze–thaw cycles is predicted and compared with the available experimental results. Figure 4b shows the variation in soil strength with increasing numbers of freeze‒thaw cycles.

It can be seen from Fig. 4b that the predicted data are basically consistent with the tested results, which reflects the deterioration law of seasonally frozen soil with the number of freeze‒thaw cycles. It is undeniable that there are some fluctuations in the middle cycles; however, this is related to the original data used for training the model and does not mean that the method is not reliable.

In summary, the PCA–ANN algorithm proposed in this study can realize the interpolation prediction of the static strength of seasonally frozen soil under unknown conditions. Although the prediction results cannot be absolutely consistent with the tested results, the deterioration law obtained for the frozen-thawed soil under different conditions is perfectly in accord with the current knowledge and related literature. One of the sources for the prediction error is that the level considered in the dataset is not large enough, which is extremely obvious in the prediction of confining pressure. Only three levels of confining pressure were considered when training the model. Furthermore, the testing procedure performed on the freeze‒thaw soil is extremely cumbersome, so there are inevitable errors in the test results. Therefore, the precision of interpolation prediction can be further improved by considering more levels of influencing factors and enhancing the experimental accuracy.

## Conclusions

In this study, three machine learning-based models, namely, the ANN, SVM and RF, for the static strength prediction of seasonally frozen soil were proposed and compared. In addition, the PCA algorithm was used to optimize the data structure so that the precision of the established model could be further improved. Finally, the established ML model was employed to predict the degradation patterns of freeze‒thaw soil. The following conclusions were drawn:

1. All three ML algorithms researched in this study have certain accuracy in predicting the static strength of frozen-thawed soil, but the ANN algorithm outperforms others, with the largest R^2^ of more than 0.95 on the validation set.
2. Based on the ANN model, the PCA algorithm was adopted to further optimize the data structure. The complexity of the model is simplified by combining the PCA and ANN algorithms, and the prediction accuracy of this model is further improved. An R-squared (R^2^) value of 0.9 on the testing set for the PCA–ANN model demonstrates its strong generalizability.
3. Reasonable prediction results reflecting the deterioration law of the seasonally frozen soil are obtained through the PCA–ANN algorithm. There is a significant reduction in the static strength of soil after the first few freeze‒thaw cycles, and then a plateau is reached. The static strength of the frozen-thawed soil decreases considerably with increasing moisture content and enhanced compaction degree.

The size of the dataset is restricted by the limited experimental samples, so the established ML model cannot reflect all the conditions faced by seasonally frozen soil in nature. The method proposed in this paper can be integrated into an application program for public use in the future. Engineers only need to input some of the key influencing factors, and the static strength or other soil properties of the seasonally frozen soil will be reasonably forecast, which can provide a scientific reference for practical engineering.

### Supplementary Information

Supplementary Information.

## Data Availability

Data is available upon request from the corresponding author for the purpose of verifying the results in this study.
