# Supplementary material for: Principal component analysis–artificial neural network-based model for predicting the static strength of seasonally frozen soils
Source: Sci Rep. 2023 Sep 26;13:16085. doi: 10.1038/s41598-023-43462-7 (PMC10522631; doi:10.1038/s41598-023-43462-7)
Supplement: Supplementary file 1 — Supplementary Information. [file 41598_2023_43462_MOESM1_ESM.pdf]

Principal component analysis–artificial neural network-based model for predicting the static strength of seasonally frozen soils

Yiqiang Sun[1,2], Shijie Zhou[1], Shangjiu Meng[1,2,3], Miao Wang[4] & Hailong Mu[2]

[1] College of Civil Engineering and Architecture, Harbin University of Science and Technology, Harbin 150080, China

[2] Key Laboratory of Earthquake Engineering and Engineering Vibration, Institute of Engineering Mechanics, China Earthquake Administration, Harbin 150080, China

[3] School of Architecture and Civil Engineering, Heilongjiang University of Science and Technology, Harbin 150022, China

[4] College of Architecture and Civil Engineering, Heilongjiang Province Hydraulic Research Institute, Harbin 100050, China

Supplementary figures:

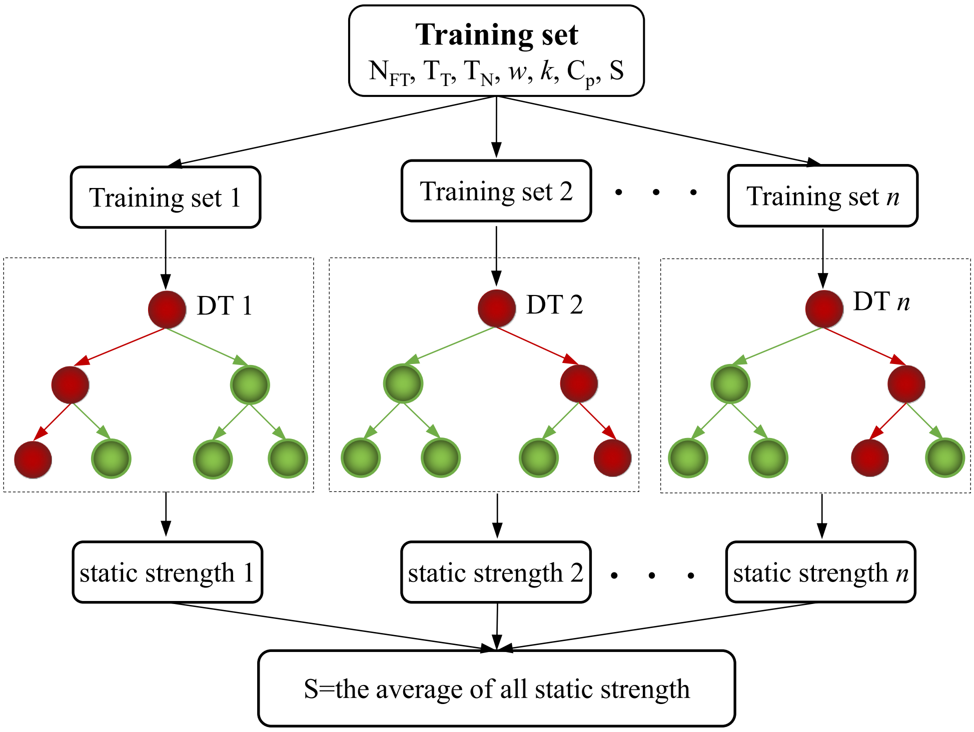

Supplementary Figure 1. Structure of the RF algorithms

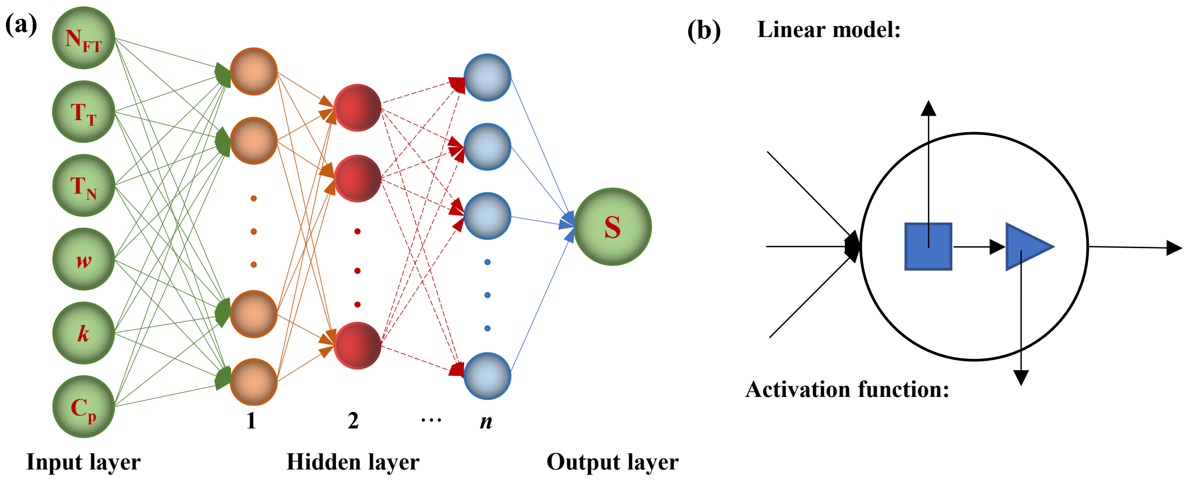

Supplementary Figure 2. Architecture of ANN algorithm:**(a)** algorithm structure; **(b)** neurons

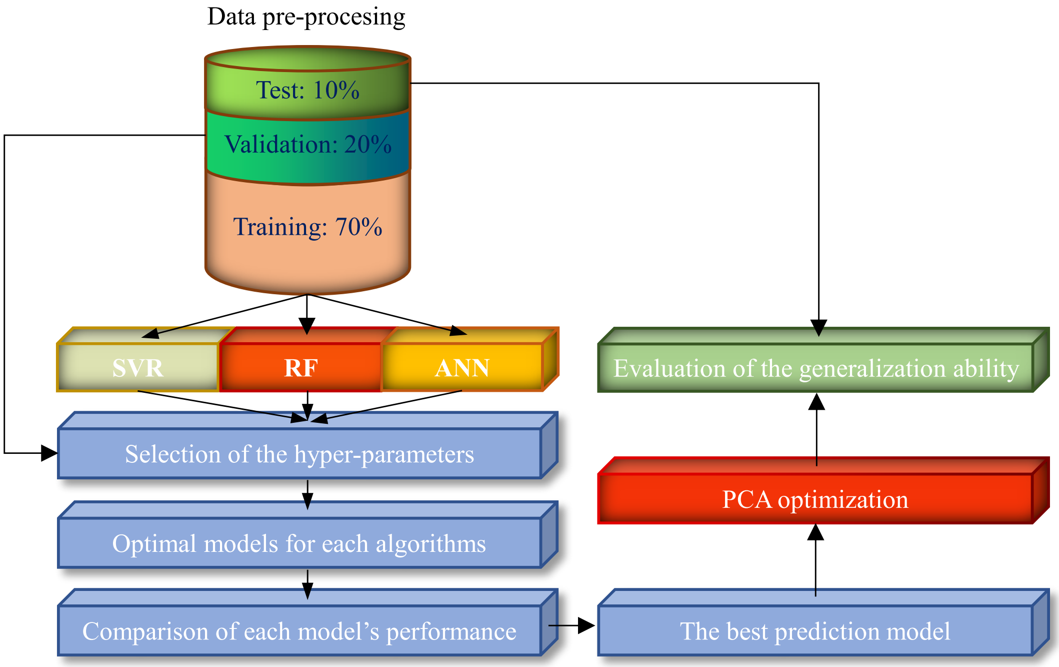

Supplementary Figure 3. Schematic view of model framework

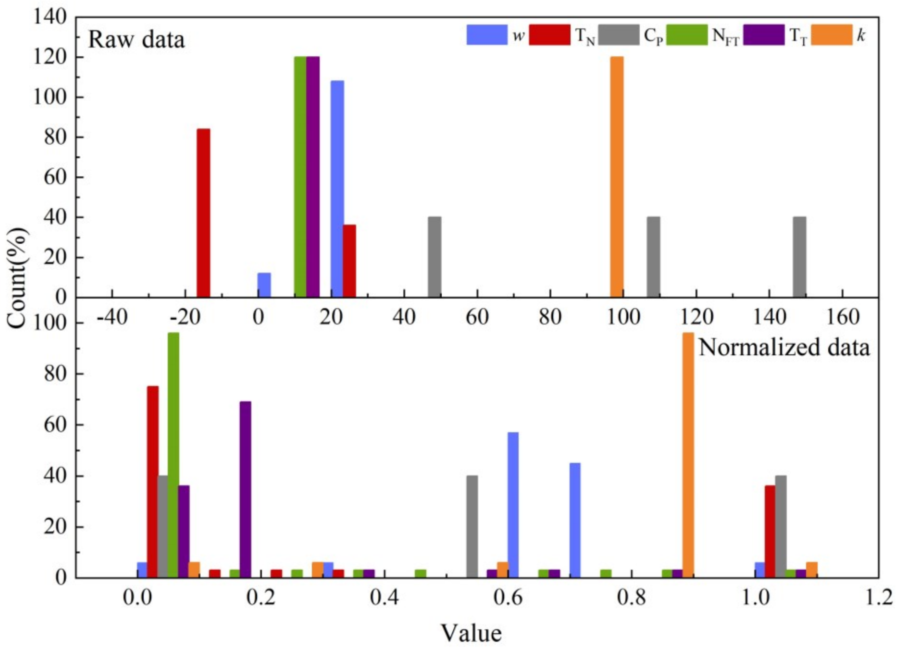

Supplementary Figure 4. Histogram of input data

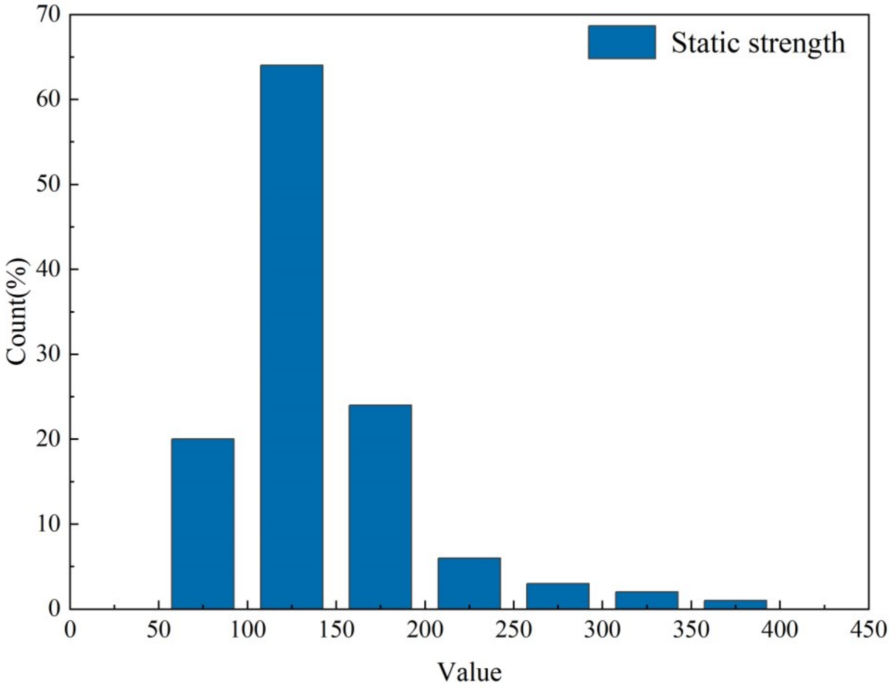

Supplementary Figure 5. Histogram of output data

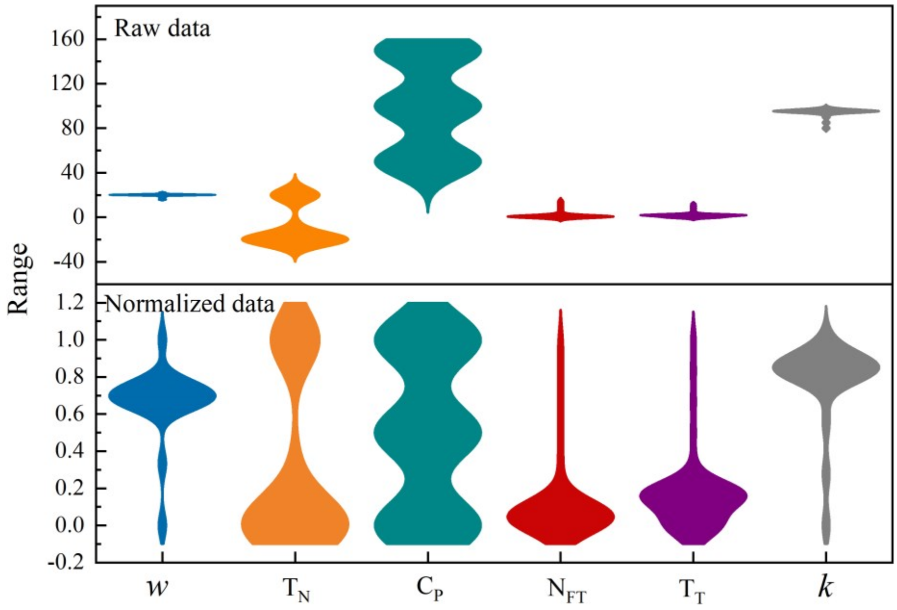

Supplementary Figure 6. Violin diagram of input data

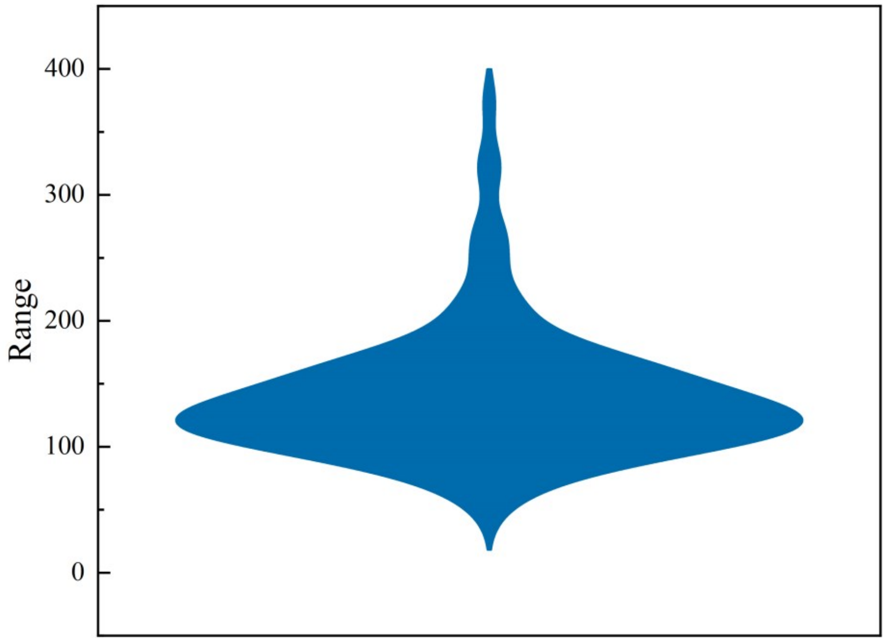

Supplementary Figure 7. Violin diagram of output data

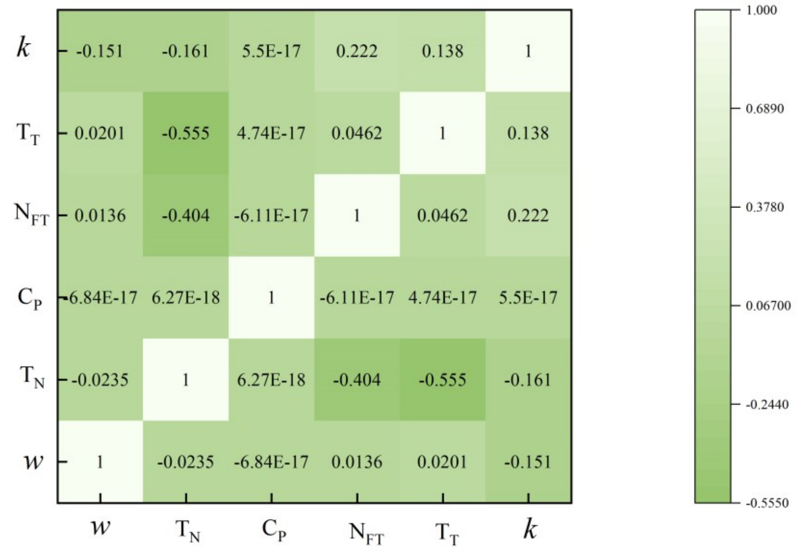

Supplementary Figure 8. Pearson Correlation Curve heat-map of data set

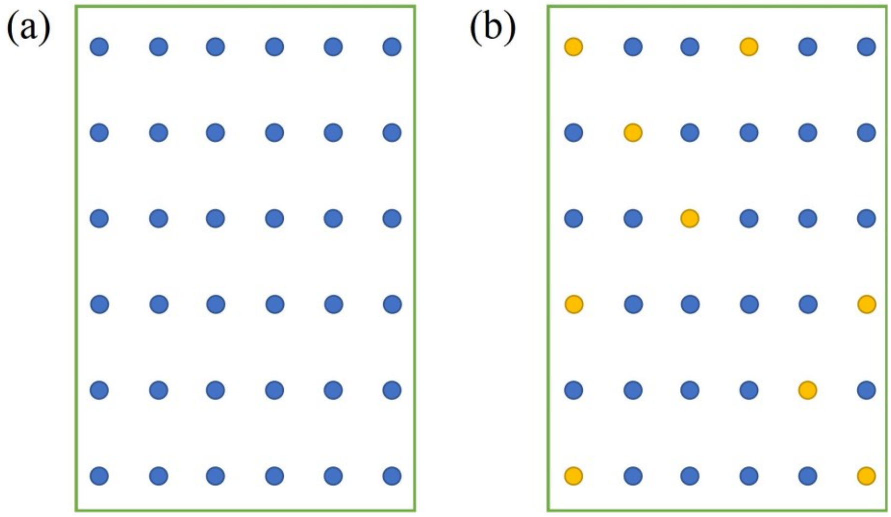

Supplementary Figure 9. Schematic view of (a) grid search and (b) random search

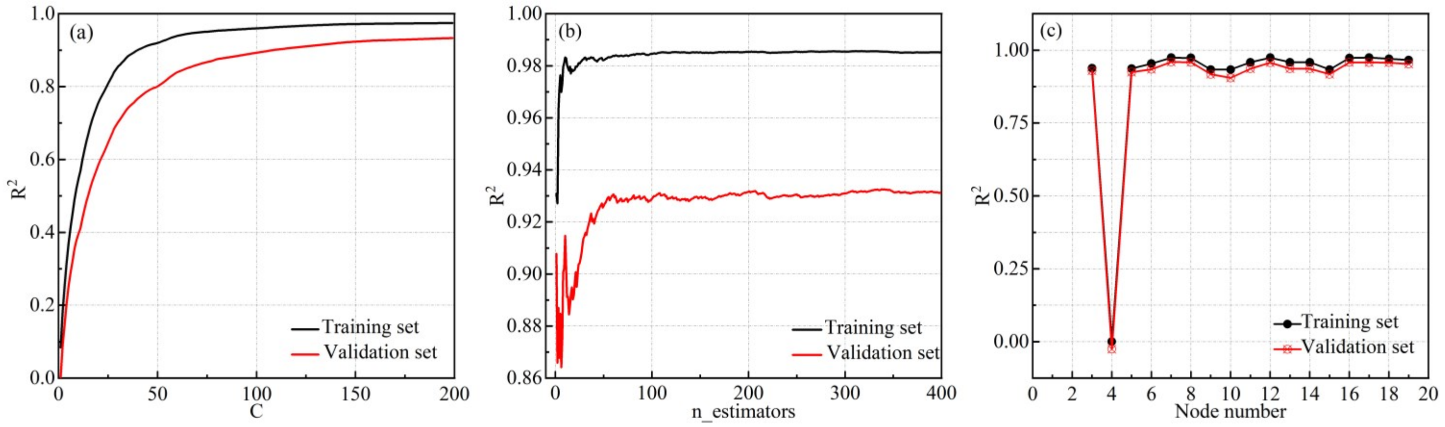

Supplementary Figure 10. Influence of hyperparameter on the Model performance: **(a)** C, **(b)** n_estimators, **(c)** number of the hidden layer

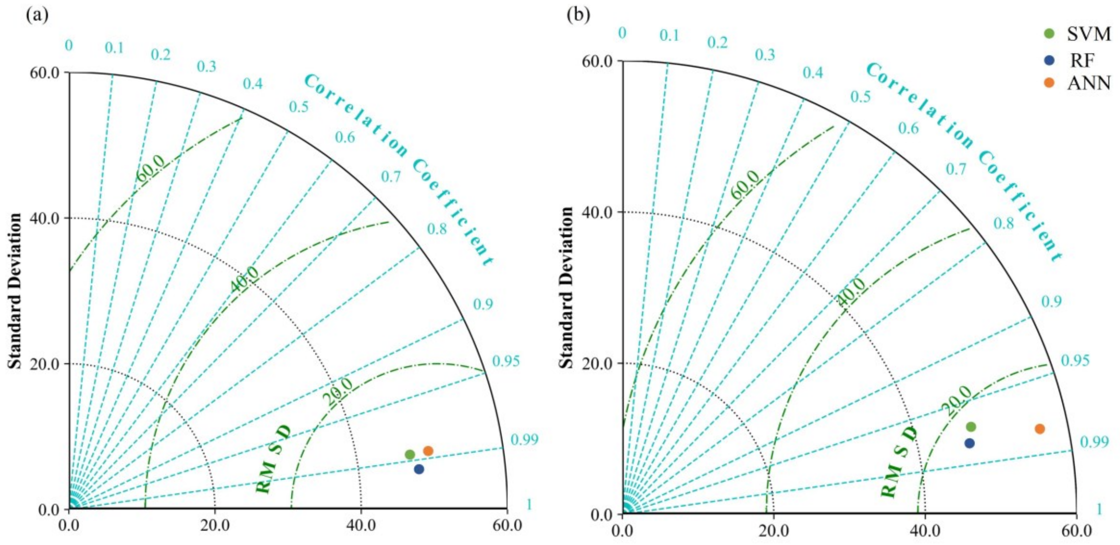

Supplementary Figure 11. Taylor diagrams of different models on the training and validation set (a)training;(b) validation.

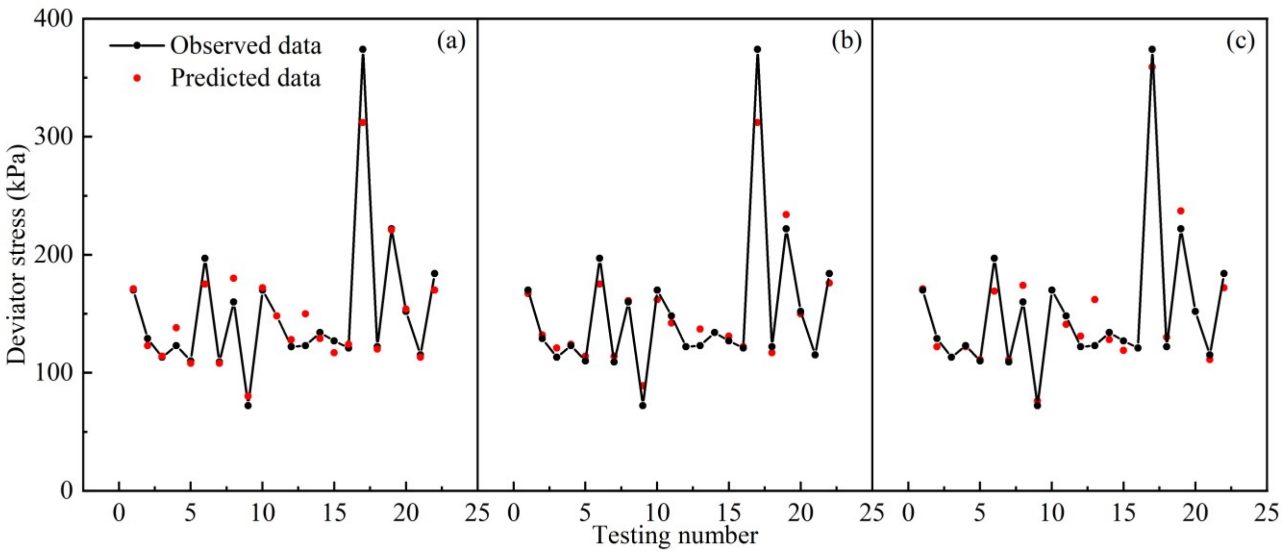

Supplementary Figure 12. Comparison between the tested and predicted results of different algorithms: **(a)** SVR, **(b)** RF, **(c)**ANN

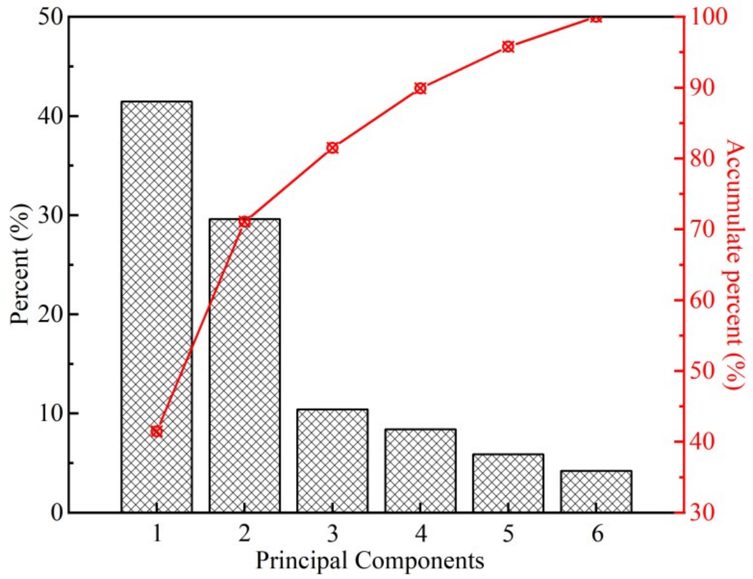

Supplementary Figure 13. Explainable variance and its cumulative value corresponding to different number of principal components

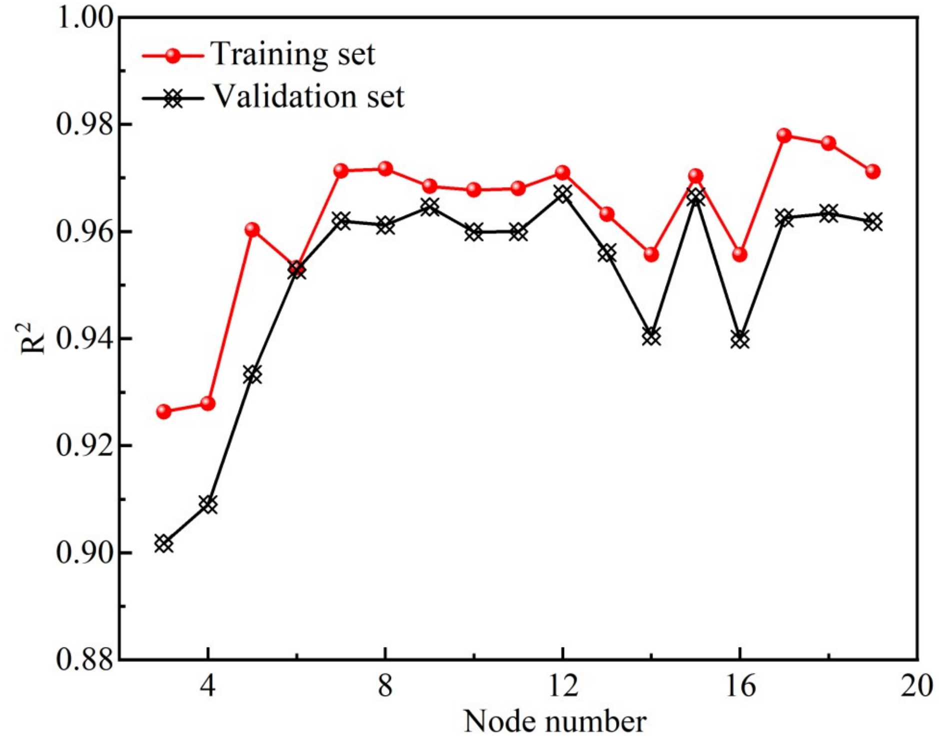

Supplementary Figure 14. Performance of models with different number of nodes in the hidden layer

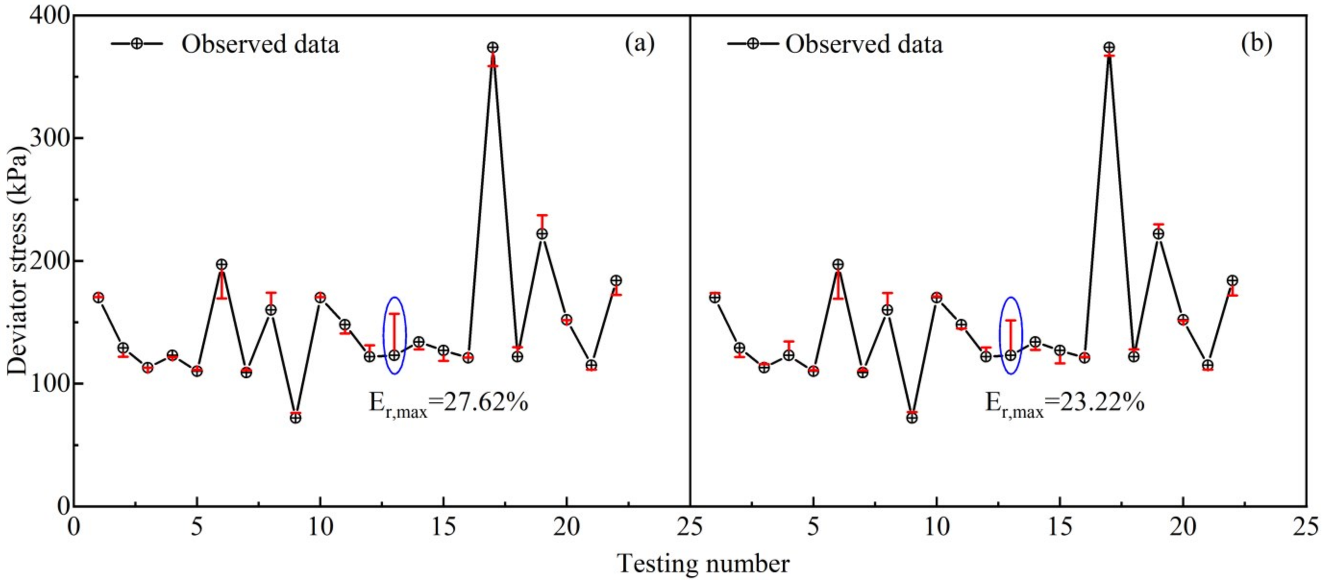

Supplementary Figure 15. Prediction accuracy before and after PCA processing: **(a)** ANN, **(b)** PCA-ANN

Supplementary tables:

Full forms	Abbreviations
Machine learning	ML
Support vector machine	SVM
Random forest	RF
Artificial neural network	ANN
Principal component analysis	PCA
Decision tree	DT
Evolutionary polynomial regression	ERF
Static strength	S
Freeze-thaw cycles	N_{FT}
Thawing time	T_T
Negative temperature	T_N
Water content	w
Compaction degree	k
Confining pressure	C_P
Stochastic Gradient Descent	sgd
Adaptive Moment Estimation	adam
Limited-memory Broyden-Fletcher-Goldfarb-Shanno Shanno	lbfgs
Coefficient of determination	R^2
Mean absolute error	MAE
Mean square error	MSE
Root mean square error	RMSE
Variance Accounted For	VAF

Supplementary Table 1. The table of abbreviations

Dataset Types	w	T_N	C_P	N_{FT}	T_T	k	S

Training data	20	-20	150	1	8	95	168
	20	-20	100	1	2	95	134
	20.5	-20	50	1	2	80	50
	20.5	-20	100	1	2	80	67
	20.4	-20	50	1	2	95	112
	20.5	20	50	0	0	85	95
	20.5	20	150	0	0	90	143
	20	-20	100	1	8	95	153
	20.5	20	100	0	0	90	131
	20	-20	50	9	2	96	98
	20.5	-20	150	1	2	90	108
	20	-20	150	7	2	96	121
	20	-20	100	1	2	96	116
	20.5	20	50	0	0	98	160
	20.5	20	150	0	0	80	98
	22	20	150	0	0	95	128
	22	-20	100	1	2	95	89
	16	20	50	0	0	95	252
	20.5	-20	50	1	2	85	65
	20	-20	50	1	2	95	117
	20.4	-5	100	1	2	95	135
	20	-20	50	5	2	96	102
	20.4	-10	100	1	2	95	132
	20.5	20	50	0	0	95	145
	22	-20	50	1	2	95	83
	20.4	20	100	0	0	95	173
	18	-20	50	1	2	95	166
	20	-20	50	11	2	96	99
	22	20	50	0	0	95	116
	20.5	20	50	0	0	90	118
	20.5	-20	50	1	2	95	97
	20	-20	150	1	4	95	163
	16	20	100	0	0	95	327
	20	-20	150	15	2	96	122
	20	-20	50	1	8	95	134
	20	-20	50	7	2	96	101
	20	-20	100	9	2	96	114
	20.4	-15	150	1	2	95	144
	20.4	20	50	0	0	95	153
	20	20	50	0	0	95	179
	20	-20	100	5	2	96	116
	22	20	100	0	0	95	124
	20.5	-20	100	1	2	95	112
	20	-20	150	13	2	96	123
	20.5	-20	50	1	2	98	107
	20	-20	150	2	2	96	122
	20.5	20	150	0	0	85	121

	20	-20	150	1	6	95	168
	20	-20	150	1	2	95	158
	20	-20	150	11	2	96	122
	20.4	-15	50	1	2	95	114
	16	-20	100	1	2	95	275
	22	-20	150	1	2	95	93
	20	-20	50	1	4	95	132
	20	-20	50	1	10	95	136
	20	-20	100	1	2	95	142
	20	-20	50	15	2	96	98
	18	-20	100	1	2	95	200
	20.5	20	150	0	0	95	163
	20.5	20	100	0	0	80	88
	20	-20	100	1	12	95	149
	20.5	20	100	0	0	85	110
	20	20	150	0	0	95	216
	20	-20	50	3	2	96	102
	20.5	20	150	0	0	98	175
	20.5	-20	50	1	2	90	80
	20	-20	150	1	2	95	144
	20	-20	100	1	6	95	149
	20	-20	100	2	2	96	113
	20	-20	150	1	12	95	170
	20.5	-20	100	1	2	85	82
	20	20	50	0	0	95	154
	20	-20	50	1	2	96	104
	20.5	20	100	0	0	95	156
	18	20	50	0	0	95	207
	20	20	50	0	0	96	144
	18	20	100	0	0	95	246
	20	20	150	0	0	95	185
	20.4	-10	150	1	2	95	142
	18	20	150	0	0	95	269
	20.4	-20	150	1	2	95	143
	20	-20	150	5	2	96	122
	20	20	150	0	0	96	166
	20.5	-20	150	1	2	98	131
	20	20	100	0	0	95	175
	16	-20	150	1	2	95	318
Validation data	20	-20	150	1	10	95	170
	20.4	-15	100	1	2	95	129
	20.4	-10	50	1	2	95	113
	20	-20	150	3	2	96	123
	20	-20	100	11	2	96	110
	20	20	100	0	0	95	197
	20	-20	100	13	2	96	109
	20	20	100	0	0	96	160

	20.5	20	50	0	0	80	72
	20.5	20	100	0	0	98	170
	20.4	-5	150	1	2	95	148
	20.5	-20	150	1	2	95	122
	20	-20	150	1	2	96	123
	20	-20	50	1	6	95	134
	20.4	-20	100	1	2	95	127
	20	-20	150	9	2	96	121
	16	20	150	0	0	95	374
	20.5	-20	100	1	2	98	122
	18	-20	150	1	2	95	222
	20	-20	100	1	10	95	152
	20	-20	100	7	2	96	115
	20.4	20	150	0	0	95	184
Testing data	20.5	-20	100	1	2	90	96
	20.4	-5	50	1	2	95	119
	20	-20	50	2	2	96	103
	20	-20	100	1	4	95	148
	20.5	-20	150	1	2	80	76
	20	-20	50	1	12	95	137
	20	-20	50	13	2	96	99
	20	-20	100	15	2	96	110
	20	-20	100	3	2	96	117
	20.5	-20	150	1	2	85	93
	20	-20	50	1	2	95	126
	16	-20	50	1	2	95	211

Supplementary Table 2 Data set

Features	unit	min	max	Number of levels	SD	median	skewness
Water contents	%	16	22	4	1.13	20	1.1
Compaction degree	%	80	98	5	4.1	95	-2.4
Confining pressure	kPa	50	150	3	41.0	100	0
Freezing	℃	-20	20	5	18.1	-20	0.81
Number of freeze-	1	0	15	10	3.6	1	2.4
Thawing time	h	0	12	7	2.6	2	2.3
Static strength	kPa	50	374		51.5	127.5	1.9

Supplementary Table 3 Data set evaluation index

Method	C	Gamma
Random Grid Search	1-200	1-100
Grid Search	50-150	0-1

Supplementary Table 4. Scope of the search for C and Gamma

MSE		MAE		RMSE		R^2	
Training set	Validation set	Training set	Validation set	Training set	Validation set	Training set	Validation set
RF	ANN	RF	ANN	RF	ANN	RF	ANN
ANN	RF	SVM	RF	ANN	RF	ANN	RF

SVM	SVM	ANN	SVM	SVM	SVM	SVM	SVM

Supplementary Table 5. Model ranking diagram of utilized indices (Values increase from top to bottom)

pseudo-codes:

```
pca=PCA(n_components=5)
pca=pca.fit(x)
x_dr=pca.transform(x)
pca.explained_variance_
pca.explained_variance_ratio_
pca.explained_variance_ratio_.sum()
pca_line=PCA().fit(x)
pca_line.explained_variance_ratio_
np.cumsum(pca_line.explained_variance_ratio_)
plt.plot([1,2,3,4,5,6],np.cumsum(pca_line.explained_variance_ratio_))
plt.xlabel("number")
plt.ylabel('variance')
plt.show
dnn2=DNN(  activation='relu'
          ,max_iter=10000
          ,random_state=420
          ,solver='lbfgs'
          ,hidden_layer_sizes=(12,)
         ).fit(x1train,np.ravel(y1train))
score=dnn2.score(x1test,np.ravel(y1test))
score
model = dnn2
def model_function(inputs):
    return model.predict([inputs])[0]
problem = {
    'num_vars': 5,
    'names': ['x1', 'x2', 'x3', 'x4', 'x5'],
    'bounds': [[-1, 1], [-1, 1], [-1, 1], [-1, 1], [-1, 1]]
}
param_values = saltelli.sample(problem, 500)
output_values = np.array([model_function(inputs) for inputs in param_values])
Si = sobol.analyze(problem, output_values)
```
